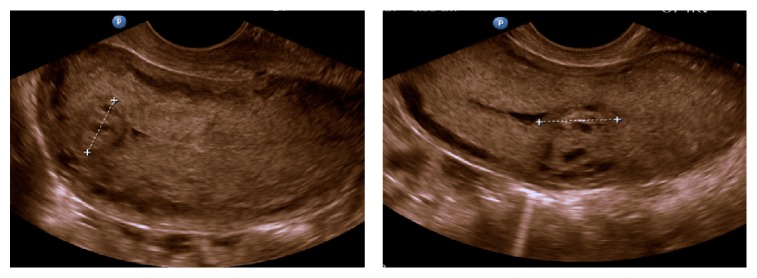# Incidental Finding of Persistent Hydatidiform Mole in an Adolescent on Depo-Provera

**DOI:** 10.1155/2016/6075049

**Published:** 2016-12-25

**Authors:** Olukayode Akinlaja, Rebecca McKendrick, Zineb Mashak, May Nokkaew

**Affiliations:** ^1^Department of Obstetrics and Gynecology, University of Tennessee College of Medicine, Chattanooga, TN, USA; ^2^University of Tennessee Health Science Center, Memphis, TN, USA

## Abstract

Molar pregnancies represent an uncommon yet important obstetric problem with potentially fatal outcomes. Patients typically present with signs and symptoms of early pregnancy, and physicians most often suspect nonmolar pregnancy complications initially; however a hydatidiform mole should be included in the differential diagnosis of a woman with a positive pregnancy test and abnormal vaginal bleeding irrespective of the use of contraception. Our case is that of an adolescent female on Depo-Provera injectable contraceptive with increased vaginal bleeding, abdominal pain, nausea, and vomiting who was incidentally found to be pregnant and subsequently diagnosed with a molar pregnancy despite persistent denial of having initiated sexual intercourse. Though gestational trophoblastic disease is uncommon with an incidence of about 1-2 cases per 1,000 pregnancies, a clinician has to display a high index of suspicion when dealing with patients at extremes of age in order to avoid potentially life-threatening outcomes.

## 1. Introduction

A hydatidiform mole is the result of an aberrant fertilization event and the most common form of a related group of lesions due to abnormal placental trophoblast proliferation known as gestational trophoblastic disease. Two types of molar pregnancies exist that are distinct in their karyotype, histopathology, gross morphology, clinical presentation, and malignant transformation risk [1, 2]. Complete moles are diploid, and about 80% are homozygous 46XX, resulting from the duplication of a single haploid sperm after fertilization of an ovum devoid of maternal chromosomes while approximately 20% maybe 46XX or XY due to dispermic fertilization of a single ovum [1, 3]. They are androgenetic as all the genetic materials are of paternal origin [4]. A complete mole is composed of hydropic chorionic villi and hyperplastic trophoblasts that lack embryonic development and, on gross inspection, resemble grape clusters due to the dilated villi.

An increased hCG level, usually >100,000 mIU/ml, is associated with complete moles and this tends to have a higher risk of malignant sequelae [5, 6].

Partial moles are triploid, 69XXX, 69XXY, or 69XYY, the product of two haploid sperms fertilizing one normal ovum. They generally have a lower hCG level than complete moles and embryonic development occurs in association with the trophoblastic hypertrophy, while the gross morphology is nonspecific and diagnosis is sometimes confirmed on pathologic review of the specimen [7].

Patients with both types of molar pregnancies typically present with symptoms of missed menstrual period, positive pregnancy test, vaginal bleeding, pelvic pain, an enlarged uterus, or hyperemesis gravidarum [8, 9].

Associated risk factors for a molar pregnancy are prior history of molar pregnancy and extremes of maternal age [10].

The diagnosis is supported by an abnormally elevated hCG and transvaginal ultrasound demonstrating a central heterogeneous mass with many discrete anechoic space, more commonly known as “the snowstorm or Swiss cheese” appearance. Both partial and complete moles are nonviable and therefore require surgical uterine evacuation. Weekly hCG measurements are then needed to demonstrate if persistent trophoblastic disease exists.

The diagnosis of postmolar gestational trophoblastic neoplasia is made based on one of the International Federation of Gynecology and Obstetrics (FIGO) criteria: hCG levels plateau over a three-week period, weekly hCG levels increase across three values recorded over at least a two-week period, persistence of detectable hCG > 6 months after uterine evacuation, or a pathologic diagnosis of choriocarcinoma [11].

Complete molar pregnancies have a 15–20% chance of developing GTN while a partial mole has approximately a 1–5% probability [12, 13].

An adolescent African American female with no significant past medical history presented with increased vaginal bleeding and abdominal pain for over a week duration and, despite repeated denials of having initiated sexual intercourse, had a positive pregnancy test and was subsequently diagnosed with gestational trophoblastic disease.

## 2. Case Presentation

A 13-year-old African American female presented to her primary care physician with vaginal bleeding and abdominal pain for a week and a half and nausea and vomiting for one month.

She had received Depo-Provera intramuscular injections for two consecutive months and attributed the bleeding to it.

A urine pregnancy test done was found to be positive and she was referred for an ultrasound confirmation by her PCP with follow-up scheduled for the next morning. Later that evening the patient reported to the emergency room with increased abdominal pain and heavy vaginal bleeding. On examination, she had a temperature of 98.1°F, blood pressure of 149/85 mmHg, pulse of 70 beats/minute, and respiratory rate of 16 cycles/minute.

Physical exam was notable for pallor, bilateral lower quadrant abdominal tenderness, a blood stained perineum, and 150 cc of clots and grape-like materials were expressed on bimanual exam.

**Figure 1:**
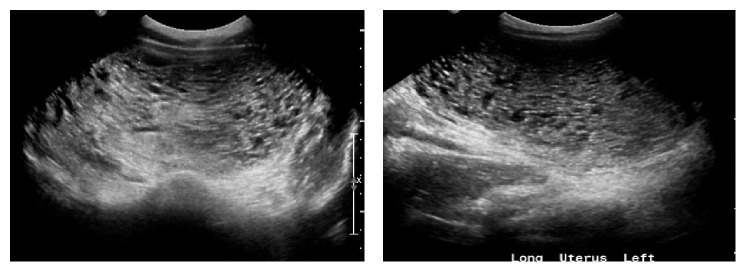


Lab tests showed a white blood cell count of 19.7, a hemoglobin of 9.3 g/dl, hematocrit of 28.6%, and beta hCG > 225,000 mIU/ml. A repeat beta hCG was 229,816 mIU/ml and a transvaginal ultrasound demonstrated an abnormally enlarged uterus filed with multicystic echogenic material as in Figure 1. The uterus was enlarged at 16.0 × 9.0 × 13.4 cm and there was a fluid-filled cervical canal devoid of an intrauterine gestation.

A molar pregnancy was diagnosed and suction dilation and curettage was performed to evacuate the uterine contents after informed consent had been obtained. Pathology report showed portions of deep red and grayish purple tissue measuring 5.0 × 3.6 × 1.2 cm in aggregate dimensions with grossly identifiable placental tissue and ovoid clusters of semitranslucent tissue while fetal tissues were not grossly evident.

A p57 immunostain was performed and demonstrated loss of expression in the syncytiotrophoblasts characteristic of a complete hydatidiform mole.

She had a negative chest radiograph and was discharged home with instructions to return to her PCP in one week for quantitative beta hCG while the need for serial monitoring and close follow-up was emphasized.

Quantitative beta hCG measurements were obtained weekly after suction D&C. At week one the level was 13,987 mIU/ml, at week 2, it was 2,254 mIU/ml, and 2,177 mIU/ml at week 3.

At her 4th week visit, her beta hCG was 1,790 mIU/ml and she complained of some occasional pelvic cramping but denied vaginal bleeding or discharge, cough, or difficulty breathing but mentioned that she had received another dose of Depo-Provera for contraception at her PCP.

A transvaginal ultrasound performed showed an enlarged uterus with boggy myometrium, peripheral hypervascularity, and a circumscribed area of tissue at the uterine fundus with demonstrable active blood flow on Doppler that was suggestive of possible retained products of contraception or residual molar disease as shown in Figure 2. After a discussion of the findings with both the patient and her mother, the decision was made to proceed with a hysteroscopy with dilation and curettage.

Operative findings comprised a 12-week size uterus and retained products of conception at the fundus of the uterus but pathological evaluation of the curetted specimen showed no residual chorionic villi.

Subsequent follow-up visits revealed undetectable beta hCG level.

## 3. Discussion

Though uncommon with approximately 1-2 cases per 1000 pregnancies, a hydatidiform mole is an important obstetric complication with potential life-threatening outcomes and thus one must have a high index of clinical suspicion for early diagnosis and prompt treatment [14].

Physicians must especially have a higher index of suspicion when dealing with patients at the extremes of maternal age as studies have demonstrated that though most molar pregnancies occur within the reproductive ages of 19–34 years, adolescents (<20 years old) are seven times more likely to develop a molar pregnancy and women of advanced maternal age (>40 years old) are nearly twice as likely [15, 16].

Complete moles are more common in both adolescents and women of advanced maternal age and, due to the associated high hCG, women with complete moles are more likely to experience the effects of hCG stimulation and have a 15–20% chance of developing persistent trophoblastic disease. Prompt diagnosis, treatment, and serial hCG monitoring are of utmost importance to prevent the development of serious sequelae of molar pregnancies.

A high index of clinical suspicion was needed to make the diagnosis in our patient, as she was an adolescent female on contraception, who had initially denied initiating sexual intercourse.

The Depo-Provera injection that she was on is about 99% effective in preventing pregnancies when injected once every three months [17].

Her vaginal bleeding could have been attributed to Depo-Provera induced irregular menstruation [17, 18].

Suction curettage as was implemented in this case is the preferred method for uterine evacuation irrespective of the size of the uterus [19] and gross inspection along with pathological evaluation of the specimen is often needed to differentiate between complete and partial molar pregnancies while immunohistochemistry staining does demonstrate absence of p57 in complete moles as a result of the paternal only genome [20, 21].

A recently completed gynecologic oncology group trial of preemptive 2nd curettage in lieu of chemotherapy in patients with low-risk nonmetastatic gestational trophoblastic neoplasia demonstrated a cure in 40% of patients [22].

Though there is a higher risk of malignant sequelae with a complete mole, both types of molar pregnancies need close monitoring after uterine evacuation as poorer gestational trophoblastic neoplasia outcomes due to advanced disease can be a consequence of poor postmolar surveillance [23].

The American College of Obstetricians and Gynecologists recommends serial hCG testing weekly until being nondetectable for 3 weeks and then monthly for 6 months [24].

Our patient was diagnosed with a complete molar pregnancy confirmed by her first operative pathology and the slow regression of her hCG levels along with the suspicious ultrasound findings led to the performance of the hysteroscopy with dilation and curettage and though her second operative pathology was benign, it is necessary to be overcautious in these situations to prevent malignancy and metastases. This scenario should be differentiated from the persistently low hCG level in the absence of clinical or radiological evidence seen in quiescent GTN [25, 26].

## 4. Conclusion

Hydatidiform mole, an uncommon complication of pregnancy, must be included in the differential diagnosis in any woman of reproductive age that presents with vaginal bleeding. This diagnosis should particularly be considered in adolescents or women of advanced maternal age, as they are more likely to have a molar pregnancy than women of normal reproductive age.

Furthermore, a molar pregnancy should not be eliminated from the differential diagnosis based on the claim of contraceptive usage, as our patient was receiving an effective contraceptive method and still developed a molar pregnancy.

After uterine evacuation, patients need an effective form of contraception and serial hCG measurements to monitor persistent trophoblastic disease while a plateauing hCG level should raise suspicion for gestational trophoblastic neoplasia and prompt intervention, as it did in our patient.

## Figures and Tables

**Figure 2:**